# Atypical Substrates of the Organic Cation Transporter 1

**DOI:** 10.3390/biom12111664

**Published:** 2022-11-09

**Authors:** Kyra-Elisa Maria Redeker, Ole Jensen, Lukas Gebauer, Marleen Julia Meyer-Tönnies, Jürgen Brockmöller

**Affiliations:** 1 Institute of Clinical Pharmacology, University Medical Centre Göttingen, 37075 Göttingen, Germany; 2 Department of General Pharmacology, Institute of Pharmacology, Centre of Drug Absorption and Transport (C-DAT), University Medical Centre Greifswald, 17487 Greifswald, Germany

**Keywords:** OCT1, SLC22A1, organic cation transporter, substrates, genetic polymorphisms

## Abstract

The human organic cation transporter 1 (OCT1) is expressed in the liver and mediates hepatocellular uptake of organic cations. However, some studies have indicated that OCT1 could transport neutral or even anionic substrates. This capability is interesting concerning protein-substrate interactions and the clinical relevance of OCT1. To better understand the transport of neutral, anionic, or zwitterionic substrates, we used HEK293 cells overexpressing wild-type OCT1 and a variant in which we changed the putative substrate binding site (aspartate474) to a neutral amino acid. The uncharged drugs trimethoprim, lamivudine, and emtricitabine were good substrates of hOCT1. However, the uncharged drugs zalcitabine and lamotrigine, and the anionic levofloxacin, and prostaglandins E2 and F2α, were transported with lower activity. Finally, we could detect only extremely weak transport rates of acyclovir, ganciclovir, and stachydrine. Deleting aspartate474 had a similar transport-lowering effect on anionic substrates as on cationic substrates, indicating that aspartate474 might be relevant for intra-protein, rather than substrate-protein, interactions. Cellular uptake of the atypical substrates by the naturally occurring frequent variants OCT1*2 (methionine420del) and OCT1*3 (arginine61cysteine) was similarly reduced, as it is known for typical organic cations. Thus, to comprehensively understand the substrate spectrum and transport mechanisms of OCT1, one should also look at organic anions.

## 1. Introduction

The organic cation transporter 1 (OCT1; SLC22A1) is most abundantly expressed in the liver [1]. Here, OCT1 is embedded in the hepatocytes’ basolateral membrane, contributing to hepatic uptake of numerous drugs, toxins, and endogenous substrates [2,3,4,5,6,7].

**Figure 1:**
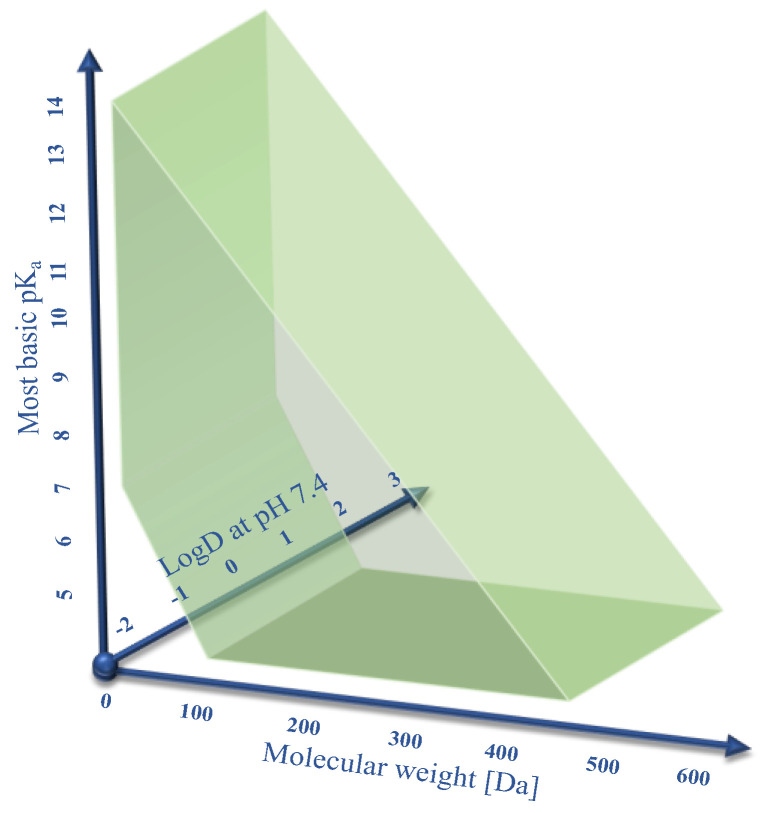
Illustration of the three primary parameters defining OCT1 substrates: a pK_a_ value above 7, a molecular weight below 500 Da, and a logD at pH 7.4 below 1.5. Here, we studied if there are also OCT1 substrates beyond these limits with a particular focus on the charge of the substrates. In this respect, neutral substances, and particularly negatively charged substances, are atypical substrates, but also zwitterionic substances are not typical substrates of OCT1.

Human OCT1 enhances the membrane transport of several organic cations. The substrate spectrum is rather diverse and includes endogenous substances and drugs with a wide range of quite different molecular structures, but most share particular features. OCT1 substrates are mostly organic substances that are protonated at physiological pH of 7.4 and have a relatively small size of up to 500 Da [6,8]. The transport is powered by membrane potential and the concentration gradient of molecules [3,9]. As illustrated in Figure 1, typical substrates of OCT1 have a basic pK_a_ above 7.4, a molecular weight below 500 Da, and are not too lipophilic, with a LogD_7.4_ below 1.5. Correspondingly, all substrates or presumed substrates that are more lipophilic, have a higher molecular weight, or have a lower basic pK_a_ are atypical substrates.

Within OCT1, the negatively charged amino acid aspartate at position 474 (Asp474), located in transmembrane helix 11, has been identified to contribute to substrate binding. Therefore, Asp474 is assumed to play a crucial role in the substrate selectivity of OCT1 and in substrate affinity [10,11]. On the other hand, if this were a critical mode of substrate binding to OCT1, OCT1 should not be able to bind substrates that are exclusively negatively charged [12]. Furthermore, binding of uncharged substrates via electrostatic forces would be less likely.

In addition to the aspartate474-to-asparagine (D474N) exchange, which we artificially introduced to probe the putatively most important negatively charged binding site, we also tested two naturally occurring inherited OCT1 variants which are found relatively frequently in many human populations and which confer significantly reduced transport activity: the methionine420-deletion (M420del; *2) and the arginine61-to-cysteine (R61C; *3) substitution [13].

Overall, organic cations are well-established substrates of OCT1 that can be translocated over a membrane. Also, some zwitterionic substrates such as isobutyrylcarnitine are considered to be substrates of OCT1 [14]. However, according to otherwise well-established transport assays, it is controversial whether OCT1 really catalyses influx or efflux transport of isobutyrylcarnitine or if the relationship between isobutyrylcarnitine and OCT1 activity is more complex [15]. Therefore, here we also tested some other zwitterionic substances as possible OCT1 substrates. Another interesting question in this context is if Asp474 also impacts the transport of non-cationic substances. If this is true, it might mean that this amino acid plays other functions in the transport process beyond substrate binding.

**Figure 2:**
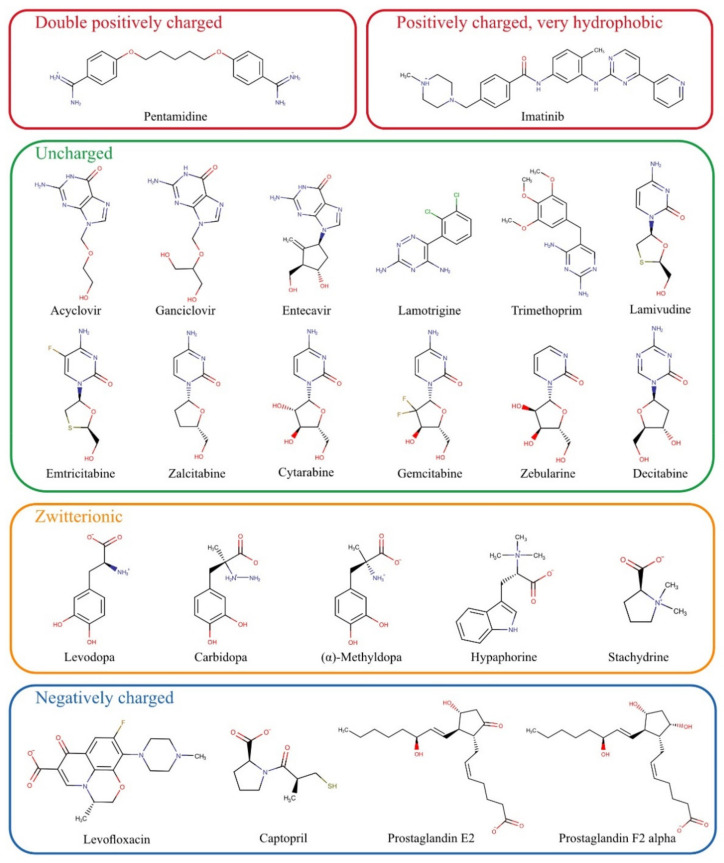
Chemical structures of potential substrates of OCT1 investigated in this study grouped by their charge at physiological pH.

For the present study, numerous substances that do not match the scheme of known OCT1 substrates were chosen based on previously published data and summarised in Figure 2. First, we tested all those substances which are entirely or mostly uncharged at pH 7.4. These include acyclovir, ganciclovir, entecavir, lamotrigine, trimethoprim, and the cytidine analogs lamivudine, emtricitabine, zalcitabine, cytarabine, gemcitabine, zebularine, and decitabine. Acyclovir and ganciclovir were identified as OCT1 substrates by Takeda, et al. [16]. OCT2 has been shown to catalyse membrane transport of entecavir [17]. Lamivudine has been identified as a substrate of OCT1 by several groups [18,19], as have lamotrigine [20] and zalcitabine [18]. Cellular influx transport of trimethoprim was classified not to be OCT1-mediated, but trimethoprim was shown to be an inhibitor of OCT1 [18]. Emtricitabine has been excluded as a substrate of OCT1 [21]. However, because of its structural similarity with lamivudine, we tested its transport via OCT1 again.

The zwitterionic substances levodopa, carbidopa, L-(–)-α-methyldopa, hypaphorine, and stachydrine have not been tested as substrates of OCT1 so far. They were chosen based on structural similarities, and their potential hepatotoxic effects would make them medically relevant if they were substrates of the hepatic transporter OCT1.

Previous data on the double positively charged substance pentamidine showed equivocal results [18,22]. Concerning the molecular interactions, pentamidine might be an exciting substrate because with its two widely spaced positive charges, it might bind simultaneously to two substrate binding sites in OCT1. Other similar substrates such as chlorhexidine were only inhibitors of OCT1 [7].

Among the exclusively negatively charged presumed OCT1 substrates, the most prominent examples were the prostaglandins E2 and F2α [12]. However, prostaglandin E2 could not be confirmed as a substrate of OCT2 [23]. The negatively charged levofloxacin has not been identified as an OCT1 substrate thus far, but the strong inhibitory effect of almost all fluoroquinolone [24] suggested that fluoroquinolones might be OCT1 substrates as well.

Two other parameters characterising atypical substrates of OCT1 are high lipophilicity and high molecular weight. Based on existing data, we also looked at whether there are OCT1 substrates that are highly lipophilic. Imatinib is positively charged and thus a possible OCT1 substrate, but it is very lipophilic and thus far, only very few, if any, very lipophilic substrates of OCT1 have been identified [25].

In summary, we wanted to explore the substrate spectrum of OCT1 beyond those substrates which are typical according to positive charge and low or moderate lipophilicity [6]. These experimental data should validate whether some neutral or even negatively charged substances can be transported by OCT1 or by its close homologs OCT2 and OCT3. From a molecular point of view, we also looked at the role of the negatively charged Asp474 in the context of negatively charged or neutral substrates, and we analysed the effects of two common naturally occurring amino acid variants of OCT1 on its transport activity with atypical substrates. Using the same assay conditions for all substrates, we wanted to compare the transport activities of well-established and controversial substrates of OCT1 quantitatively. In the course of these experiments, we also identified some new substrates.

## 2. Materials and Methods

### 2.1. Test Substrates

Most substrates used for this project were obtained from Sigma-Aldrich (Taufkirchen, Germany). The internal standard buformin was from Wako (Osaka, Japan), acyclovir from Adooq Bioscience (Irvine, CA, USA), cytarabine, prostaglandin E2 and F2α from Biomol (Hamburg, Germany), decitabine and zebularine from Absource (München, Germany), hypaphorine from Biorbyt (Cambridge, UK), L-(–)-α-methyldopa from APExBIO (Houston, TX, USA), ranitidin-d6 from Toronto Research Chemicals (Toronto, ON, Canada), and zalcitabine was from ChemScene (Monmouth Junction, NJ, USA).

### 2.2. Cell Lines

The transport experiments were performed using HEK293 cells that overexpress human OCT1*1 (wild-type), mutant human OCT1 D474N, variant human OCT1*2 (M420del) and OCT1*3 (R61C), wild-type human OCT2, and OCT3 after stable transfection. The wild-type cell lines of OCT1 and OCT2, as well as the variant cell lines OCT1*2 and *3, were generated using the Flp-In system (Thermo Fisher Scientific, Darmstadt, Germany) as described in previous publications [13,26,27]. The cell line overexpressing OCT3 was a kind gift from Dr. Koepsell and Dr. Gorbulev (University of Würzburg, Germany). Cells transfected with the empty pcDNA5/FRT vector were used for the transport experiments as a control cell line. All cells were cultured approximately up to passage 30.

The introduction of the point mutation D474N into pcDNA3.1::hOCT1 was performed by site-directed mutagenesis. For this purpose, a polymerase chain reaction (PCR) was performed consisting of 2.5 µL 10 × KOD buffer (KOD Hot Start DNA Polymerase Kit; Merck, Darmstadt, Germany), 5 µL Q-solution (Qiagen, Hilden, Germany), 2.5 µL dNTPs (each 2 mM; Thermo Fisher Scientific, Darmstadt, Germany), 1 µL MgSO_4_ (25 mM; Merck, Darmstadt, Germany), 1 µL DNA (50 ng/µL), 0.5 µL HotStart KOD polymerase (KOD Hot Start DNA Polymerase Kit; Merck, Darmstadt, Germany), 11.2 µL double-distilled water, and 0.65 µL of both forward and reverse primer containing the point mutation (D474N-forward primer: GTGTGTTCCTCCCTGTGTAACATAGGTGGGATAATCACC; D474N-reverse primer: GGTGATTATCCCACCTATGTTACACAGGGAGGAACACAC). The PCR started with 3 min at 95 °C followed by 19 cycles of 30 s denaturation at 95 °C, 30 s primer annealing at 70 °C and 4 min elongation at 72 °C before the PCR was finished by cooling down to 8 °C. Afterwards, the reaction mixture was digested with the methylation-specific restriction enzyme DpnI to remove the template plasmid. For this purpose, the PCR product was incubated with 2 µL DpnI at 37 °C. After 1 h, an additional 1 µL DpnI was added to the mixture and was incubated for another hour at 37 °C. Then, the digested PCR product was prepared for transformation via electroporation into OneShot TOP10 electrocompetent *E. coli* (Thermo Fisher Scientific, Darmstadt, Germany). After validation of the correct sequence, the mutated transporter OCT1 D474N was cloned into the vector pcDNA5/FRT (Thermo Fisher Scientific, Darmstadt, Germany).

Finally, stable transfection of HEK293 T-Rex cells with the mutated transporter OCT1 D474N was also achieved using the Flp-In system (Thermo Fisher Scientific, Darmstadt, Germany). HEK293 T-REx cells were seeded on 6-well plates with 1 × 10^6^ cells/well 24 h in advance. As preparation for transfection, 100 µL pure DMEM (Dulbecco’s Modified Eagles Medium) was mixed with 400 ng pcDNA5 plasmid DNA and 3.6 ng pOG44 helper plasmid; in a second tube, 100 µL pure DMEM was mixed with 12 µL FuGene6 transfection reagent. After 5 min of pre-incubation, the first tube containing the DNA was transferred to the second tube containing the transfection reagent, mixed by pipetting, and incubated for 15 min at room temperature. Meanwhile, cells were washed with DMEM medium containing 10% fetal bovine serum (FBS) once before they were covered with 2 mL of it for further steps. The transfection mixture was added dropwise onto the cells and the cells were incubated for 24 h in the incubator before the medium was switched to DMEM supplemented with 10% FBS and penicillin/streptomycin. A further 24 h later, the cells were transferred to a petri dish and were allowed to attach to the dish overnight before 300 µg/mL Hygromycin B was added. Only the cells that integrated and expressed the plasmid with hygromycin B resistance successfully were able to survive. The cell culture medium was renewed after four to five days. The cells were given ten days past hygromycin treatment to form colonies derived from single cells which were transferred to a 24-well plate (with DMEM medium containing 10% FBS, P/S and 50 µg/mL hygromycin B) and further to a 6-well plate and T25 cell culture flasks.

**Figure 3:**
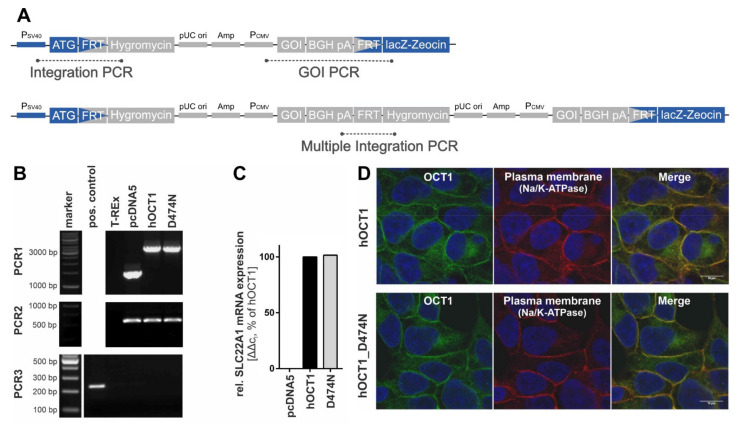
Validation of HEK293 cells expressing variant OCT1 D474N. (**A**). Scheme of the plasmid with its integrated gene and the target position of the three PCR products. (**B**). The three validation PCRs show the presence of the gene-of-interest (PCR1, 3137 bp), the integration of the plasmid (PCR2, 519 bp), and the exclusion of multiple integrations (PCR3, 214 bp). (**C**). The gene expression of the variant was determined via quantitative real-time PCR at one early cell line passage and is shown in comparison to the wild-type. (**D**). The immunofluorescence staining shows the subcellular localisation of OCT1 wild-type and variant using confocal microscopy (magnification 100×) with co-staining of OCT1 (green) and Na^+^/K^+^-ATPase (red).

The validation of the correct genomic integration of the plasmid was performed with three independent PCRs as described previously [13,26] (Figure 3A,B). Firstly, by the verification of the presence of the gene-of-interest (GOI), secondly, by the successful integration of the plasmid, and thirdly, by the exclusion of multiple integrations. The gene expression analysis was performed with a quantitative real-time PCR at an early cell line passage (Figure 3C). The subcellular localisation was shown with immunofluorescence staining that was imaged using a Laser Scanning Microscope LSM710 (Carl Zeiss Microscopy GmbH, Oberkochen, Germany) (Figure 3D). The primary antibodies that were used were a monoclonal mouse anti-human OCT1 (2C5) (range of immunogen used: amino acid 284–347) (Novus Biologicals, Abingdon, UK) and a monoclonal rabbit anti-Na^+^/K^+^-ATPase (EP1845Y) (Abcam, Cambridge, UK). The corresponding secondary antibodies were the polyclonal Alexa Fluor^®^ 488 goat anti-mouse IgG (H + L) and Alexa Fluor^®^ 546 goat anti-rabbit IgG (H + L) (Thermo Fisher Scientific, Darmstadt, Germany).

### 2.3. In Vitro Cellular Uptake Experiments

The HEK293 cells were cultured in DMEM medium with D-Glucose (4.5 g/L), L-Glutamine (584 mg/L), and pyruvate (110 mg/L) that was supplemented with 10% (*v*/*v*) FBS, and the antibiotics penicillin (100 U/mL) and streptomycin (100 µg/mL).

Forty-eight hours before the transport experiments, 600,000 cells were plated on 12-well plates pre-coated with poly-D-lysine and incubated at 37 °C with 5% CO_2_. The prepared 12-well plate with cells was positioned on a heating plate pre-warmed to 37 °C. After removing the medium, the cells were washed with 37 °C HBSS+ (10 mM HEPES in Hank’s balanced salt solution, pH 7.4). Then, one pre-defined concentration of a substrate was added to each well. The incubation period was stopped by adding 2 mL of 4 °C HBSS+ and removing the cells from the heating plate. The cells were washed twice with 4 °C HBSS+. For cell lysis, the cells were incubated for 15 min with 500 µL of 80% acetonitrile (LGC Standards, Wesel, Germany), which contained a respective internal standard (Table 1).

For time-dependent transport, the cells were incubated with 1 µM of the substance for 15 to 240 s to determine the linear uptake range ≥ 15 s. For concentration-dependent transport, the cells were incubated with defined substrate concentrations (0.1, 1, 10, 30, 100, 300, 600, 1000 µM) for the respective pre-defined incubation times. If no complete concentration-dependent uptake experiments were performed, cellular uptake ratios were determined using two distinct concentrations of the substances (1 µM; 100 µM) and pre-defined incubation times. The uptake by a transporter was normalised to the uptake by empty vector-transfected cells. The categorisation of substrates and non-substrates based on uptake ratios was implemented with a ratio cut-off of 2, as suggested by the FDA. The results of transport experiments were normalized to the total protein concentration per well. Total protein quantification was performed using a bicinchoninic acid assay with bovine serum albumin as standard.

### 2.4. HPLC-MS/MS Concentration Analyses

All intracellular drug concentrations were analysed by HPLC-MS/MS using a Shimadzu Nexera 2 UHPLC system with an LC-30AD pump, an SIL-30AC autosampler, a CBM-20A communication module and a CT0-20AC column oven, and an online degassing module. The HPLC separation was conducted at a temperature of 40 °C and with a flow of 300 µL/min for all substances. The stationary phase was a Brownlee SPP RP-Amide column with an inner dimension of 4.6 × 100 mm and a particle size of 2.7 µm protected with a Phenomenex C18 pre-column. The mobile phase of the HPLC contained 0.1% (*v*/*v*) formic acid and a certain proportion of organic additive consisting of acetonitrile:methanol 6:1 (*v*/*v*) which was individual for each substance (Table 1).

The substances of interest were detected with an API 4000 triple quadrupole mass spectrometer using the parameters listed in Table 1. The analytes solved in 0.1% formic acid were quantified by using the Analyst software (Version 1.6.2, AB SCIEX, Darmstadt, Germany) with the parameters stated in Table 1. Calibration was performed with known substrate concentrations.

### 2.5. Calculations

The ratios of cellular uptake were calculated by dividing the total uptake of transporter-overexpressing cells by the total uptake into empty vector transfected cells. The concentration-dependent cellular net uptake was used for analysis of transport kinetics by subtracting for each substrate concentration the uptake of empty vector-transfected cells from the uptake of transporter-overexpressing cells. The transport kinetic parameters V_max_ and K_M_ were determined with non-linear regression by using the Michaelis–Menten equation for net uptake data and by using the Michaelis–Menten equation extended with a linear term for total uptake data (GraphPad Prism 5.01, GraphPad software, LA Jolla, CA, USA). The intrinsic clearance Cl_int_ was expressed with the ratio of V_max_ and K_M_.

## 3. Results

### 3.1. Screening of Atypical Substrate Candidates for OCT1-Mediated Transport

**Figure 4:**
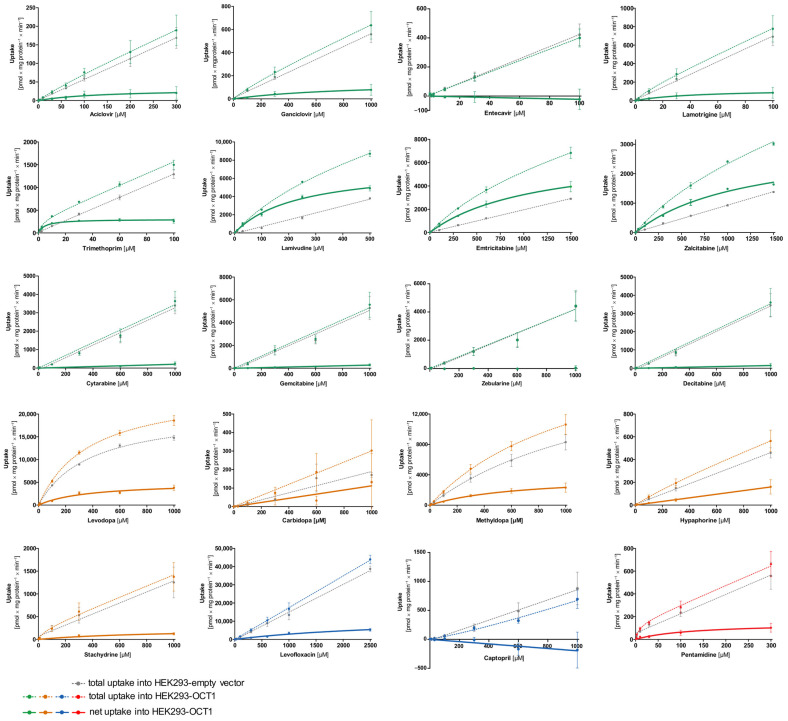
Concentration-dependent uptake of potential substrates of OCT1. HEK293 cells that were transfected stably with the OCT1 transporter were incubated with the candidate substrates. The total uptake of various concentrations of the substances within the time range indicated in Table 1 by OCT1 (coloured–dotted curve) and empty vector overexpressing HEK293 cells (grey–dotted curve) are shown. The bold curve shows the net uptake which is calculated by subtraction of the total uptake by empty vector-transfected cells from the total uptake by OCT1-transfected cells (coloured–straight curve). The data points are displayed as means ± SEM. The experiments were conducted at least three times (n ≥ 3) independently from each other for every substance.

First, transport kinetic constants of several selected atypical substances by OCT1 were determined (Figure 4, Table 2). A categorization of substrates using the intrinsic clearance was implemented with a cut-off of >5 µL/mg protein/min for good substrates and an intrinsic clearance between 1–5 µL/mg protein/min for poor substrates. Substances with an intrinsic clearance below <1 µL/mg protein/min or for which cell uptake was not significantly above the values found in empty vector-transfected cells were classified as non-substrates of OCT1.

As illustrated in Figure 4, the concentration-dependent total uptake of acyclovir and ganciclovir increased essentially linearly with a rising substrate concentration for the OCT1 and empty vector cell lines. Although net uptake resembled a hyperbolic curve, the marginal difference between the two cell lines indicates only very poor OCT1-mediated transport (Figure 4). This observation is also reflected in the kinetic parameters for acyclovir and ganciclovir with a V_max_ of 31.2 and 146 pmol/mg protein/min, respectively, as well as a K_M_ value of 142 and 849 µM, respectively, leading to extremely low intrinsic clearances (Acyclovir 0.22 µL/mg protein/min; Ganciclovir 0.17 µL/mg protein/min). Thus, OCT1-mediated transport cannot be considered as relevant for both substrates. This is also the case for entecavir, which is not a substrate of OCT1 based on the linear course of the concentration-dependent curve of both cell lines (OCT1 and empty vector) with nominally even higher intracellular concentrations in the empty vector-transfected cells.

Lamotrigine was identified as a poor substrate of OCT1. Due to the low intracellular concentrations, V_max_ and K_M_ could only be determined with a relatively large error (Table 2). In contrast to lamotrigine, trimethoprim, lamivudine, and emtricitabine were transported efficiently with intrinsic clearances of 62.9, 31.4, and 6.15 µL/mg protein/min, respectively, whereas the zalcitabine showed only poor transport efficiency because of a high K_M_ value (Table 2).

As shown in Figure 2, cytarabine, gemcitabine, zebularine, and decitabine are structurally similar to the OCT1 substrates emtricitabine, lamivudine, and zalcitabine. However, despite the structural resemblance, none of the four antimetabolites showed uptake by OCT1 (Figure 4). 

The net uptake of levodopa and methyldopa by OCT1 indicates a saturated concentration-dependent uptake with intrinsic clearances of 13.8 and 5.94 µL/mg protein/min, respectively. However, both substrates also showed saturable concentration-dependent uptake into the empty vector-transfected cells with V_max_ values of 20,681 and 488 pmol/mg protein/min and K_M_ values of 380 and 32.2 µM (intrinsic clearances for empty vector-transfected HEK cells of 54.4 and 15.1 µL/mg protein/min, respectively). In conclusion, levodopa and methyldopa might be transported by OCT1 to a small extent, however, not as efficiently as some of the neutral substances (such as lamivudine), and further proteins of HEK cells might transport levodopa and methyldopa as well.

Carbidopa and hypaphorine show a linear increase in total uptake of both cell lines (OCT1 and empty vector) where saturation cannot be observed within the studied concentration range. Similar to acyclovir, stachydrine also shows only a minimal difference between the uptake of the two cell lines, which indicates only very poor OCT1-mediated transport.

Pentamidine represents, with its double positive charge (allowing it to possibly bind to two of the OCT1 substrate binding sites), another kind of atypical substrate candidate. Here, a saturation curve of net uptake by OCT1 can be observed. Michaelis–Menten analysis predicts an intrinsic clearance of 1.15 µL/mg protein/min which is very low, and therefore, it can be considered a poor substrate of OCT1.

**Figure 5:**
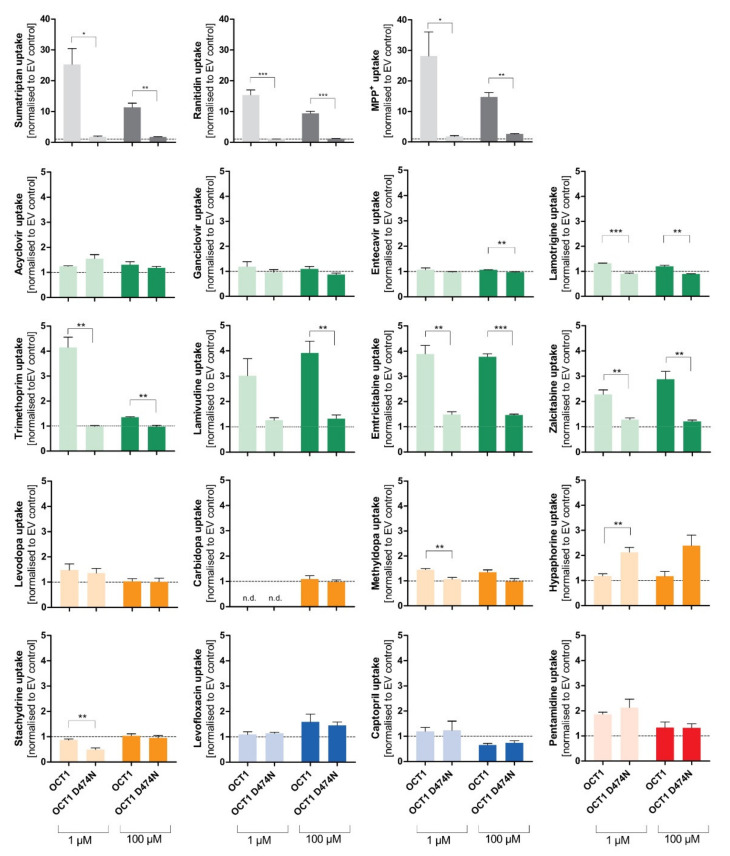
Effect of the D474N variant on transport of typical and atypical substrates of OCT1. HEK293 cells that were transfected stably with the OCT1 transporter, as well as cells overexpressing the variant OCT1 D474N (aspartate474-to-asparagin), were incubated with 1 µM (light colour) and 100 µM (dark colour) of each substance for their substrate-specific incubation time (Table 1). The substrates are grouped based on their overall charge which is indicated by the characteristic colour. The abbreviation “n.d.” indicates that the intracellular substrate concentration was below limit of quantification (not detected). The uptake by OCT1 and its variant were normalised to the uptake by the empty vector, and zero net uptake is indicated with a dotted line. The experiments were conducted at least three times (n ≥ 3) independently from each other for every substance. Statistical significance between OCT1 and OCT1 D474N was determined using an unpaired students *t*-test with * *p* < 0.05, ** *p* < 0.01, and *** *p* < 0.001.

The net uptake of the negatively charged levofloxacin by OCT1 might indicate a minor OCT1-mediated uptake. However, the curve was not saturated at the highest substrate concentration (Figure 4), corresponding to a very high V_max_ of 12,550 pmol/mg protein/min and a K_M_ value of 3326 µM. Thus, considering the concentration-dependent transport kinetics and uptake ratios (e.g., Figure 5) levofloxacin is a poor substrate of OCT1. By contrast, captopril was not an OCT1 substrate at all. Since the concentrations in the OCT1 transfected cells were even lower than in those of the mock-transfected cells, one might conclude efflux transport; however, with the experimental error, we conclude that there was no effect.

### 3.2. Effect of the D474N Variant on Transport of Typical and Atypical Substrates of OCT1

The negatively charged aspartate474 in OCT1 is thought to play an important role in substrate binding. However, with neutral or acidic substrates it is questionable how binding to this site might occur. Therefore, next we tested the influence of the negatively charged aspartic acid at position 474 (Asp474). For this purpose, the OCT1 cell line with a mutation at position 474 from aspartate to asparagine (OCT1 D474N) was used. The impact of the absent negative charge was tested for all substrate candidates, as well as for three well-established cationic substrates of OCT1 (sumatriptan, ranitidine, and 1-methyl-4-phenylpyridinium (MPP^+^)), to observe an altered uptake by OCT1 D474N compared to OCT1 wild-type (WT) (Figure 5).

The uptake of the cationic substrates sumatriptan, ranitidine, and MPP^+^ by the OCT1 variant was significantly decreased 20- to 30-fold compared to the wild-type transporter. This confirmed the assumption that Asp474 plays a role in substrate binding and transport. Trimethoprim, emtricitabine, and lamivudine, that had been classified as good OCT1 substrates according to the concentration-dependent transport assays, but also some poor OCT1 substrates including lamotrigine and zalcitabine, revealed the same tendency as seen for the excellent OCT1 substrates: the uptake by OCT1 D474N is significantly reduced by a factor of between 2 and 4. Although the baseline value of uptake by OCT1 wild-type was much lower compared to the cationic substrates, a significant reduction in uptake by the OCT1 D474N variant could still be observed.

Unexpectedly, the exchange of the acid aspartate to the neutral asparagine resulted in a two-fold enhancement of hypaphorine uptake. This effect was seen in all replicate measurements (*p* < 0.01). Apparently, with hypaphorine a more efficient transport occurs because of the missing negatively charged amino acid Asp474. In contrast to the linear uptake by wild-type, concentration-dependent uptake by the OCT1 variant indicated a saturable uptake with a V_max_ of 850 pmol/mg protein/min and K_M_ of 1573 µM.

### 3.3. Effect of the Two Natural OCT1 Variants on Transport of Typical and Atypical Substrates of OCT1

In addition to the artificial variant, the two naturally occurring variants OCT1*2 (methionine420del) and OCT1*3 (arginine61cysteine) were also used to test the uptake of atypical substrates. Both variants demonstrate a reduced uptake of cationic OCT1 substrates, and the experiments aim to investigate the impact of the altered amino acids on atypical substrates.

**Figure 6:**
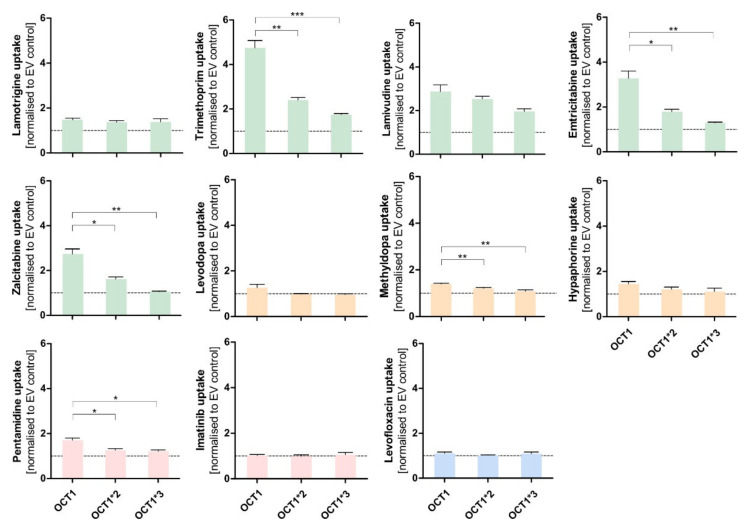
Effect of the naturally occurring OCT1 variants *2 and *3 on the transport of atypical substrates of OCT1. HEK293 cells that were transfected stably with OCT1*1 as well as with the variants OCT1*2 (M420del) and OCT1*3 (R61C). All these cell lines were incubated with 1 µM of the shown substances for their substrate-specific incubation time (Table 1). The substrates are grouped based on their overall charge which is indicated by the characteristic colour. The uptake by OCT1 and its variants were normalised to the uptake by the empty vector, and a zero net-transport rate is indicated with a dotted line. The experiments were conducted at least three times (n ≥ 3) independently from each other for every substance. Statistical significance between OCT1 and its variants was determined using an unpaired students *t*-test with * *p* < 0.05, ** *p* < 0.01, and *** *p* < 0.001. No adjustment for multiple testing was performed because every comparison should be considered as a separate experiment.

The expected reduction in uptake by both variants could be observed for several of the tested substrates, such as trimethoprim, lamivudine, emtricitabine, zalcitabine, and even pentamidine to a small extent (Figure 6). The very lipophilic substance imatinib did not show uptake by OCT1.

### 3.4. Comparison of Transport via OCT1, OCT2, and OCT3

**Figure 7:**
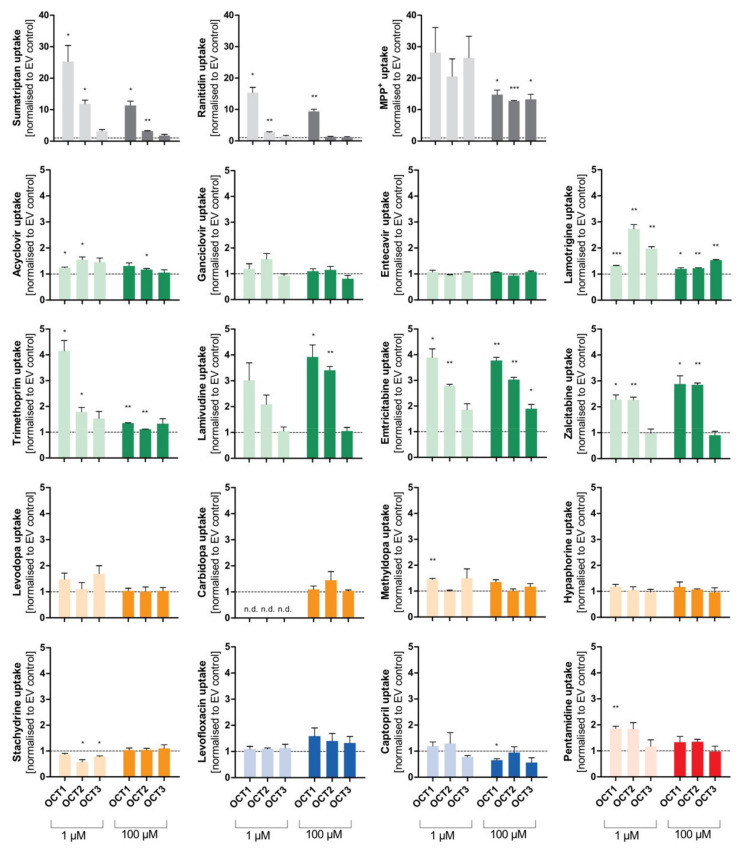
Comparison of transport via OCT1, OCT2, and OCT3. Cellular uptake ratios were performed with HEK293 cell lines that were transfected stably with the OCT1, OCT2, and OCT3 transporters, respectively, and that were incubated with 1 µM (light colour) and 100 µM (dark colour) of each substance for their specific incubation time (Table 1). The abbreviation “n.d.” indicates that the intracellular substrate concentration was below limit of quantification (not detected). The uptake by OCT1, OCT2, and OCT3 was normalised to the uptake by the empty vector, and zero net transport is indicated with a dotted line. The experiments were conducted at least three times (n ≥ 3) independently (i.e., in different experiments) for every substance. Statistical significance between empty vector-transfected cells and OCT1/2/3, respectively, was determined using a one-sample *t*-test comparing each of the transport activities of the 3 transporters and the tested substrates with the ratio of 1.0 meaning zero net transport (* *p* < 0.05, ** *p* < 0.01, and *** *p* < 0.001). Thus, we did not ask whether there were significant differences between the 3 transporters, but only whether or not the transport rates for each transporter were significantly different from one.

The uptake of the selected atypical substrate candidates was additionally analysed for the closely related cation transporters OCT2 and OCT3. Although the substrate characteristics of OCT2 and OCT3 are similar to OCT1, differences in the substrate spectrum may still exist (Figure 7).

Aside from the established substrates sumatriptan, ranitidine, and MPP^+^, that show uptake ratios of up to 30 by OCT1, the selected substances emtricitabine, lamivudine, zalcitabine, and trimethoprim (at 1 µM) demonstrate uptake by OCT1 with ratios between 2 and 4. Similarly, cellular uptake by OCT2 was observed for emtricitabine, lamivudine, and zalcitabine, and for lamotrigine (at 1 µM). Concerning OCT3, only the well-known substrates sumatriptan (at 1 µM) and MPP^+^ showed a relevant uptake. Influx of lamotrigine and emtricitabine was slightly more rapid with OCT3 than with mock-transfected cells, but the ratios were below 2, which is a frequently used cutoff ratio for relevant transport.

### 3.5. Prostaglandins as Substrates of OCT1

Among the most striking atypical substrates of OCT1 are the prostaglandins E2 and F2α [12]. Here, we wanted to confirm this data with our cell constructs, and we wanted test the effects of the D474N variant. Since the prostaglandins are exclusively negatively charged, binding to that amino acid should not take place at all and, thus, the substitution should not affect transport rates.

**Figure 8:**
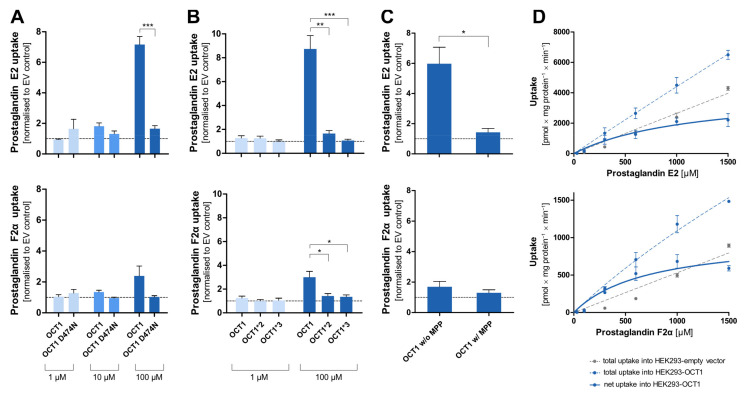
Cellular uptake of prostaglandins by OCT1. Cellular uptake ratios were determined in HEK293 cells that were transfected stably with OCT1 or its variants that were incubated with 1 µM (light blue), 10 µM (medium blue), and 100 µM (dark blue) of prostaglandin E2 and F2α for 120 s. Thus, four separate sets of experiments confirmed the carrier-mediated transport of PGE2, and three separate sets of experiments confirmed carrier-mediated transport of PGF2α. (**A**). Uptake ratio by variant OCT1 D474N in comparison to OCT1 wild-type. (**B**). Uptake ratio by natural variants OCT1*2 (M420del) and OCT1*3 (R61C) in comparison to the wild-type. (**C**). Uptake ratio of OCT1 wild-type without and with the inhibitor MPP^+^ (1 mM inhibitor, 100 µM prostaglandin). (**D**). Concentration-dependent total uptake by OCT1 WT (dotted blue curve) and empty vector (dotted grey curve) as well as the resulting net uptake (straight blue curve). The uptake by the transporters was normalised to the uptake by the empty vector. The experiments were conducted at least three times (n ≥ 3) independently with both prostaglandins. Statistical significance of the uptake ratio between OCT1 and its variants and between inhibited and not inhibited OCT1 was determined using an unpaired students *t*-test with * *p* < 0.05, ** *p* < 0.01, and *** *p* < 0.001.

Surprisingly, the negatively charged prostaglandin E2 showed strongly increased uptake by OCT1 at higher concentrations. A similar but less efficient transport was also seen with prostaglandin F2α. As it had been observed for the OCT1 substrates, the uptake by the OCT1 variant D474N, as well as of the natural variants OCT1*2 and *3 in comparison to the wild-type, was reduced to the level of the control cell line (Figure 8A,B). The transport of prostaglandin E2 can be inhibited by the potent OCT1 inhibitor MPP^+^ (1 mM) which can be seen in the statistically significantly reduced uptake of prostaglandin E2 (Figure 8C). The nominally reduced uptake of prostaglandin F2α after inhibition was not statistically significant due to a lower baseline uptake without inhibitor. The kinetic analysis (Figure 8D) revealed saturable uptake of prostaglandin E2 and F2α (V_max_ of 4119 and 1007 pmol/mg protein/min; K_M_ of 1177 and 718.8 µM, respectively). Consequently, based on the intrinsic clearances of 3.5 and 1.4 µL/mg protein/min, respectively, they can both be considered (poor) substrates of OCT1. The difference in transport caused by the protein variants, the suppression of transport by the inhibitor MPP^+^, and the concentration-dependences verified that there is indeed a carrier-mediated transport of the two prostaglandins by OCT1.

## 4. Discussion

Organic cation transporters enhance the membrane transport of organic cations, and some of our previous screening studies for OCT1 substrates have explicitly excluded non-cationic substrates [6,7]. However, other previous publications have indicated that several neutral or even anionic substances might also be substrates of cation transporters. Therefore, this study addressed the substrate specificity of OCT1 with emphasis on charge at neutral pH.

Compared to many typical positively charged substrates of OCT1, most atypical substrates were transported with moderate or very low transport rates. With several of them, OCT-mediated transport was only minimally above the transport observed in empty-vector transfected cells. Among the atypical substrates, the highest transport rates were observed for trimethoprim, lamivudine, and emtricitabine. Only moderate transport rates were confirmed, for instance, for lamotrigine, zalcitabine, and prostaglandin E2. On the other hand, no or only very poor transport was observed, for instance, for acyclovir, gancyclovir, entecavir, hypaphorine, stachydrine, and captopril (Table 3).

In general, K_M_ values were much higher than typical blood concentrations of the substrates. For instance, considering the OCT1 substrate sumatriptan, its K_M_ concentration of about 60 µM would correspond to a blood concentration of about 17,800 µg/L, which is 600-fold higher than typical human maximum blood concentrations of about 30 µg/L. Nevertheless, we see significant effects of OCT1 deficiency on human sumatriptan pharmacokinetics [28]. One explanation may be that local concentration, in this case in the liver sinusoids after oral absorption, may be much higher than systemic blood concentrations.

The antiviral acyclovir was even considered as a prototypical substrate of a specific substrate binding site in OCT1 [29]. It was tested by using a mouse-derived S2 cell line and radioactive [3H]-acyclovir [16]. They found OCT1-mediated intrinsic clearances of 0.88 and 0.388 µL/mg protein/min for acyclovir and ganciclovir, respectively. Our estimates (Table 3) were about four-fold smaller, but both publications consistently showed that these antiviral drugs are only very poor substrates of OCT1, and OCT1 is very unlikely to play a relevant role in disposition of these antivirals.

Lamotrigine had been identified as a substrate of OCT1 using the BBB-representing cell line hCMEC/D3 with a K_M_ value of 62 µM [20]. This cell line does not express relevant amounts of OCT1, and there is essentially no correlation between OCT1 activity and transport via the H^+^OC antiporter dominating influx transport in hCMEC/D3 cells [30]. As shown by our transport experiments, lamotrigine is a poor substrate of OCT1 and a slightly better substrate of OCT2 and OCT3 (Table 3, Figure 7). One SNP (OCT1*1B, rs628031) showed a moderate association, but this SNP is functionally very similar to the wild-type transporter, and thus, this genetic association cannot confirm that OCT1 plays a relevant role for lamotrigine [31].

The antibacterial drug trimethoprim is clearly transported by OCT1 and has the lowest K_M_ and highest intrinsic clearance of all selected substances (Table 2). In comparison to OCT1, the uptake by OCT2 and OCT3 was only minimal. Some influx transport of trimethoprim has already been observed by others [18]. These authors have concluded from the relatively high unspecific uptake that there is no relevant OCT1-mediated transport. However, our measurements reproducibly showed that uptake in OCT1 overexpressing cells was higher than in the empty vector-transfected cells. Still, with a pK_a_ of 7.16, trimethoprim is not strictly an atypical substrate, since it is partly positively charged at pH 7.4, and that positively charged fraction may drive transport via OCT1. Since about 75% of trimethoprim is eliminated unchanged via the kidneys, the clinical relevance of OCT1-related genetic variation or drug–drug interactions is questionable in normal kidney function. However, in significantly impaired renal function, the hepatically eliminated fraction may increase, and in this condition, patients with genetic OCT1 deficiency may have very high blood concentrations and more adverse effects.

The uptake of the antiviral drug lamivudine by OCT1 is characterised by an intrinsic clearance of 31.4 µL/mg protein/min indicating that lamivudine is transported by OCT1 relatively efficiently. This confirms data of several publications [18,19]. Although there were some differences in the transport kinetic constants between the studies, all estimates were in the same order of magnitude. Overall, it can be summarised that despite some differences in kinetic parameters, both cited publications and the data presented here have shown that OCT1 transports lamivudine and that this (at physiological pH uncharged) drug is indeed a good substrate of OCT1. Further, lamivudine is also a substrate of OCT2, confirming previous data [18]. Thus, genetic variation and interactions at OCT2 may play a role in the renal elimination of lamivudine (about 90% of lamivudine is eliminated via the kidneys). Because of the primary renal elimination, the genetic polymorphisms in OCT1 may not be relevant for the systemic clearance of lamivudine. However, lamivudine is also used in the treatment of hepatitis B. Whether or not OCT1 polymorphisms might predict therapeutic efficacy of lamivudine against hepatitis B might be an interesting research topic.

Emtricitabine, which is a cytidine analogue like lamivudine, was clearly identified as an OCT1 substrate by an uptake ratio (over empty vector-transfected cells) of around 4 and an intrinsic clearance of 6.15 µL/mg protein/min. The K_M_ value of emtricitabine was significantly lower compared to lamivudine. This might be due to the fluor group, but numbers of derivatives studied here do not allow us to draw general conclusions. No OCT1 transport of emtricitabine was found during other studies [18,21] but we have shown concentration-dependent uptake and additional uptake ratios of approximately 4 (measured at two concentrations), which clearly demonstrated uptake of emtricitabine by OCT1 as well as by OCT2.

Other structurally very similar cytidine analogues (cytarabine, gemcitabine, zebularine, and decitabine) did not show any transport by OCT1. In this context, one may ask what the difference between the substrates and non-substrates is. Firstly, the sulphur within the ring structure of lamivudine and emtricitabine could be an additional factor for efficient transport by OCT1, since it cannot be found in the structure of the other substances. Secondly, it can be observed that lamivudine and emtricitabine (as well as decitabine) have a different three-dimensional orientation due to different chiral configurations which could be an additional factor for substrate characteristics for OCT1; however, the different enantiomers were not available to test that hypothesis. Overall, it can be concluded that minor structural differences may impact the translocation by OCT1 enormously.

The zwitterionic drugs levodopa and methyldopa apparently showed some uptake by OCT1 (Figure 4). However, the saturable concentration-dependent uptake by the control cell line indicates that not only diffusion, but also transport, occurs into this cell line. Most likely, the HEK293 cells do have some basal expression of amino acid transporters such as the tyrosine transporter ASCT2 (SLC1A5), which might also contribute to cell uptake of levodopa [32]. Because of this interference, precision of the transport kinetic constants for levodopa and methyldopa were only limited and this might be the reason for the discrepancy between the intrinsic clearances and ratios, of which the latter showed only minimal uptake.

Interestingly, the zwitterionic tryptophan metabolite hypaphorine, which showed no signs of transport by OCT1, revealed a significantly increased uptake by the variant OCT1 D474N. This is in clear contrast to almost all other substrates of OCT1, which showed greatly reduced or abolished transport when the negative charge of aspartate474 was removed. Apparently, the absence of the negative charge at position 474, which was proposed to affect the selectivity of cations, promotes the binding of hypaphorine to the transporter and, consequently, has a direct impact on its transport.

In the case of the cationic and several neutral substances (trimethoprim, emtricitabine, lamivudine, lamotrigine, and zalcitabine), the D474N variant showed a reduced uptake effect. This suggests that the negative charge is important for interactions within the protein, which would be independent from the substrate. The positive charge might determine or facilitate the initial binding of a substrate to OCT1, but the translocation of a substrate might depend on further factors.

Substances with two strongly positively charged groups localized about 10 Å apart in the molecule are also non-typical substrates of OCT1, which are impressively demonstrated by the strong OCT1 inhibitor chlorhexidine and related agents [33]. In part, this might be simply explained by the unspecific detergent-like effect of these molecules. However, such substrates may bind specifically with their two positive charges to the transporter’s different substrate binding sites. Two or more different binding sites of OCT1 have been suggested by several authors [29]. The antimicrobial drug pentamidine has a similar structure and apparently was transported to some extend by OCT1 (Figure 4). Nevertheless, uptake by OCT1 was relatively small, leading to the conclusion that pentamidine is a very poor substrate of OCT1. Previously published data about pentamidine were contradictory. Whereas one group has not identified pentamidine as a substrate of OCT1 or OCT2 [18], transport studies by other scientists have classified pentamidine as an OCT1 substrate with a K_M_ value of 36.4 µM and an intrinsic clearance of 4.3 µL/mg protein/min [22]. The latter constants are similar to those obtained in the present study.

The negatively charged prostaglandins E2 and F2α are, with their pK_a_ of −1.63, far from typical OCT1 substrates. However, the high uptake ratio of prostaglandin E2, the fact that transport is inhibited by the OCT1 inhibitor MPP^+^, and the observation of a saturable uptake strongly indicate that it is transported by OCT1, which confirms previous results [12]. However, with a high K_M_ of around 1 mM, it is questionable whether this plays any medically relevant role in the systemic clearance of prostaglandins since blood concentrations of, for example, the prostaglandin E2, are in the picogram per millilitre range (nanomolar range) [34]. These very low concentrations are due to spill-over of the locally acting prostaglandins. However, OCT1 is expressed at low levels in several other tissues than liver, and in these tissues, OCT1 may contribute to the regulation of prostaglandin released there in higher concentrations.

Irrespective of the questionable physiological relevance of OCT1 for the effects of prostaglandin E2, the transport of this exclusively negatively charged molecule is interesting for our understanding of the substrate binding mechanisms and our understanding of the negative charge at aspartic acid 474 in OCT1. As shown in Figure 8A, transport of prostaglandin E2 was almost abolished by removing the negative charge at amino acid 474. At least two interpretations are possible: first, aspartate474 is not (only) relevant for transporter–substrate interactions, but also for interactions within the protein. Second, there may still be substrate interactions between the two negative charges which are then mediated by divalent positively charged organic or inorganic molecules. Further experiments are needed to confirm these speculations.

## 5. Conclusions

**Figure 9:**
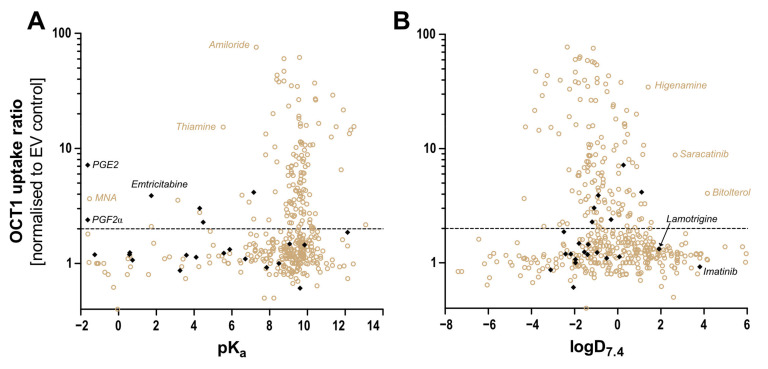
Correlation between the OCT1 uptake ratio and (**A**) pK_a_ and (**B**) logD at pH 7.4. with ratio uptake data from here presented atypical substrates (black) and from published substrates (brown) [6,7,8,35]. An uptake ratio of 2 was also indicated earlier as the cut-off between substrates and non-substrates or irrelevant substrates. As illustrated, only very few substrates with a pK_a_ below 6 are known thus far. Thiamine, with its high uptake ratio, is an exception from that rule, as is prostaglandin E2. Concerning lipophilicity, at present, there are only two substrates with a logD_7.4_ above 2 and a relevant OCT1 transport ratio.

In conclusion, several substrates that are atypical as OCT1 substrates, since they are uncharged, zwitterionic, negatively charged, or double-positively charged, were transported by OCT1. Using the same methods for transport measurement revealed a wide range of high to very low transport activities with these atypical substrates (Table 3). As shown in the context of further substrates (Figure 9), only in exceptional instances, OCT1-mediated transport does exist with substrates with a pK_a_ below 6 or a logD_7.4_ above 2. 

However, with most of these substrates, the transport efficiency is clearly lower compared to established cationic substrates. While the neutral antiviral drugs lamivudine, emtricitabine, and zalcitabine were good substrates of OCT1, other structurally similar derivates were only very poorly, or not at all, transported. Even anionic and double positively charged substances as prostaglandin E2 and pentamidine were clearly confirmed as substrates of OCT1. It might be suggested that the positive charge of substrates has an essential impact on the initial binding of substrate to transporters, but the translocation to the other side is affected by the structure of the substrate. Pharmacokinetically, all the good substrates of OCT1 identified here (trimethoprim, lamivudine, emtricitabine, zalcitabine) are mostly eliminated via the kidneys. Thus, OCT1 does not play a relevant role for their hepatic elimination, at least not in case of normal renal function. However, OCT1 may be relevant if these drugs need to enter hepatocytes to achieve their therapeutic effects. Altogether, this project has aimed at identifying several novel and atypical OCT1 substrates and, thereby, has improved our understanding of OCT1 substrate recognition.

## Figures and Tables

**Table 1:** HPLC, detection and transport assay parameters.

Substance	IS	RT [min]	Q1 Mass [Da]	Q3 Mass [Da]	DP [V]	CE [V]	CXP [V]	Incub. Time [s]
**3% organic additive** (96.9% ddH_2_O, 0.1% formic acid, 2.6% acetonitrile, 0.4% methanol)								
Buformin		4.1	158.0	60.0	40	35	10	
Cytarabine	Buformin	3.1	244.125	112.0 (95.1)	50	17 (58)	6 (18)	120
Decitabine	Buformin	4.0	229.116	113.0 (85.9)	40	11 (39)	6 (16)	120
Gemcitabine	Buformin	4.1	264.093	112.0 (95.1)	50	24 (59)	6 (18)	120
Lamivudine	Buformin	4.3	229.95	111.92 (95.01)	46	17 (53)	6 (18)	15
Levodopa	Buformin	4.0	197.89	152.07 (180.1)	41	19 (13)	15 (12)	60
L-(–)-α-Methyldopa	Buformin	5.0	212.0	195.0 (177.0)	41	9 (10)	13 (15)	120
Stachydrine	Buformin	3.4	144.006	84.0 (58.2)	70	30 (37)	16 (10)	120
Zebularine	Buformin	4.2	229.123	97.0 (78.9)	41	12 (9)	18 (14)	120
**8% organic additive** (91.9% ddH_2_O, 0.1% formic acid, 6.9% acetonitrile, 1.1% methanol)								
Acyclovir	Sotalol	3.8	225.98	151.9 (134.93)	46	17 (40)	10 (8)	120
Carbidopa	Sotalol	3.9	227.23	181.0	40	19	12	120
Emtricitabine	Ranitidin-d6	5.5	247.95	129.97	44	15	8	60
Entecavir	Ranitidin-d6	4.9	278.07	152.03 (135.04)	69	25 (50)	10 (9)	120
Ranitidin	Ranitidin-d6	4.35	315.3	176.0 (130.1)	65	24 (34)	11 (8)	120
Ranitidin-d6		4.3	321.2	176.0 (130.1)	65	25 (35)	15	
Sotalol		4.1	273.37	255.1 (133.1)	61	17 (37)	16 (8)	
Sumatriptan	Ranitidin-d6	6.4	296.2	58.2 (251.2)	50	30 (24)	12	120
Zalcitabine	Sotalol	3.1	212.1	112.0 (95.0)	41	13 (47)	6 (18)	120
**12% organic additive** (87.9% ddH_2_O, 0.1% formic acid, 10.3% acetonitrile, 1.7% methanol)								
Fenoterol		4.8	304.1	107.1 (135.2)	80	44 (24)	12	
Trimethoprim	Fenoterol	5.6	291.0	230.19 (123.01)	94	33	15 (8)	60
**20% organic additive** (79.9% ddH_2_O, 0.1% formic acid, 17.2% acetonitrile, 2.8% methanol)								
Ganciclovir	Nadolol	3.0	256.042	152.0 (135.2)	50	17 (45)	10 (8)	120
Hypaphorine	Nadolol	4.6	247.0	146.2 (188.1)	50	31 (19)	10 (12)	120
Lamotrigine	Nadolol	5.25	256.0	211.0 (187.0)	100	36 (37)	13	120
Levofloxacin	Nadolol	3.9	362.2	318.0 (261.0)	71	27 (37)	22 (18)	60
MPP^+^	Nadolol	3.5	170.016	154.0 (128.1)	100	43 (42)	10 (8)	120
Nadolol		3.5	310.06	254.1 (201.0)	66	23 (31)	16	
Pentamidine	Nadolol	3.7	341.184	324.2 (120.1)	96	27 (55)	10 (8)	60
**35% organic additive** (64.9% ddH_2_O, 0.1% formic acid, 30% acetonitrile, 5% methanol)								
Captopril	Nortriptyline	4.9	218.124	69.9	40	35	14	120
Nortriptyline		5.1	264.2	233.2 (91.1)	46	21 (30)	16 (6)	
**50% organic additive** (49.9% ddH_2_O, 0.1% formic acid, 42.9% acetonitrile, 7.1% methanol)								
Chloramphenicol		4.1	322.97	151.9 (150.8)	−75	−23 (−29)	−9	
Prostaglandin E2	Chloramphenicol	5.7	351.212	315.3 (271.2)	−75	−16 (−24)	−9 (−5)	120
Prostaglandin F2α	Chloramphenicol	5.5	353.217	309.1 (43.0)	−90	−26 (−68)	−7 (−5)	120

Abbreviations: RT, retention time; Q1/Q3 mass, first/third quadrupole mass filter with quantifier and (qualifier); DP, declustering potential; CE, collision energy; CXP, collision cell exit potential; Incub. time, incubation time in the transport experiments, chosen according to prior experiments the maximum time with linear substrate formation was chosen.

**Table 2:** Kinetic parameters of substrates OCT1.

Substrate	V_max_ (±SEM) [pmol/mg Protein/min]	K_M_ (±SEM) [µM]	Cl_int_ (±SEM) [µL/mg Protein/min]
Sumatriptan	2167 ± 243	58.9 ± 5.1	36.8 ± 7.32
Ranitidin	1047 ± 123	54.5 ± 7.2	19.2 ± 4.8
MPP^+^	2263 ± 184	108 ± 17.4	20.8 ± 5.04
Acyclovir	31.2 ± 19.2	142 ± 189	0.22 ± 0.43
Ganciclovir	146 ± 144	849 ± 1560	0.17 ± 0.49
Entecavir			
Lamotrigine	123 ± 76.2	44.4 ± 63.1	2.77 ± 5.66
Trimethoprim	300 ± 16.9	4.78 ± 1.24	62.9 ± 19.9
Lamivudine	7295 ± 582	232 ± 43.0	31.4 ± 8.31
Emtricitabine	6902 ± 1110	1123 ± 335	6.15 ± 2.82
Zalcitabine	3069 ± 280	1194 ± 193	2.57 ± 0.65
Cytarabine			
Gemcitabine			
Zebularine			
Decitabine			
Levodopa	4993 ± 884	363 ± 163	13.8 ± 8.6
Carbidopa			
Methyldopa	3843 ± 1001	647 ± 335.6	5.94 ± 4.63
Hypaphorine			
Stachydrine	238 ± 134	881 ± 910	0.27 ± 0.43
Pentamidine	143 ± 63.1	124 ± 136	1.15 ± 1.75
Levofloxacin	12,550 ± 3723	3326 ± 1458	3.77 ± 2.77
Captopril			
Prostaglandin E2	4119 ± 1415	1177 ± 744	3.50 ± 3.42
Prostaglandin F2α	1007 ± 244	7198 ± 387	1.40 ± 1.09

Note: SEM, standard error of the mean. The first three substrates are well-established good OCT1 substrates and were included into the present testing as reference. If no constants are given, transport in OCT1 overexpressing cells was not significant above transport in empty vector-transfected cells.

**Table 3:** Summary of the transport data for OCT1, OCT2, and OCT3.

Substance	Charge at pH 7.4	logD at pH 7.4	pK_a_	Cl_int_ [µL/mg Protein/min] of OCT1	OCT1	OCT2	OCT3
Sumatriptan	+1	−1.24	9.54	36.8	++	++	+
Ranitidin	+1	0.45	7.80	19.2	++	+	0
MPP^+^	+1	−1.54		20.8	++	++	++
Pentamidine	+2	−2.50	12.13	1.15	+/−	0	0
Imatinib	+1	3.80	7.84		0		
Acyclovir	0	−1.55	0.6	0.22	+/−	+/−	0
Ganciclovir	0	−2.18	0.58	0.17	0	0	0
Entecavir	0	−1.96	0.74		0	0	0
Lamotrigine	0	1.91	5.89	2.77	+/−	+	+/−
Trimethoprim	0	1.10	7.16	62.9	+	+/−	0
Lamivudine	0	−1.10	4.30	31.4	+	+	0
Emtricitabine	0	−0.90	1.74	6.15	+	+	+
Zalcitabine	0	−1.19	4.48	2.57	+	+	0
Levodopa	+1–1	−1.80	9.06	13.8	0	0	0
Carbidopa *	+1–1	−1.96	8.49		0	0	0
Methyldopa	+1–1	−1.37	9.85	5.94	+/−	0	0
Hypaphorine	+1–1	−1.40			0	0	0
Stachydrine	+1–1	−3.11		0.27	0	0	0
Levofloxacin	−1	−0.51	6.72	3.77	0	0	0
Captopril	−1	−2.42	−1.26		0	0	0
Prostaglandin E2 *	−1	0.26	−1.63	3.5	+		
Prostaglandin F2α *	−1	−0.31	−1.63	1.4	+		

Note: The dark green fields with a “++” indicate very good substrates of OCTs that have an intrinsic clearance >15 or show an uptake ratio >10. The medium green fields with a “+” mark good substrates of OCTs when the intrinsic clearance is between 5–15 or the ratio is 2–10. The lightest green fields with a “+/−” indicate poor transport with a Cl_int_ < 5 or ratio between 1–2 with statistical significance (*p* < 0.05). The symbol “0” with grey background indicates that no transport by OCT1, OCT2, and OCT3 occurred (i.e., no kinetic parameters or uptake ratio <2 without significance). The categorisation is based on the intrinsic clearance of kinetic analysis for OCT1 in column five and on the ratio uptake data at 1 µM concentration (* exception: 100 µM concentration for carbidopa, prostaglandin E2 and F2α) in column six to eight; discrepancy between intrinsic clearance and uptake ratio of OCT1 is visualised with a colour difference.

## Data Availability

The raw data supporting the conclusions of this article will be made available by the authors, without undue reservation.
